# Systematic synergy modeling: understanding drug synergy from a systems biology perspective

**DOI:** 10.1186/s12918-015-0202-y

**Published:** 2015-09-16

**Authors:** Di Chen, Xi Liu, Yiping Yang, Hongjun Yang, Peng Lu

**Affiliations:** Institute of Automation, Chinese Academy of Sciences, Beijing, 100190 China; Institute of Chinese Materia Medica, China Academy of Chinese Medical Sciences, Beijing, 100700 China

## Abstract

Owing to drug synergy effects, drug combinations have become a new trend in combating complex diseases like cancer, HIV and cardiovascular diseases. However, conventional synergy quantification methods often depend on experimental dose–response data which are quite resource-demanding. In addition, these methods are unable to interpret the explicit synergy mechanism. In this review, we give representative examples of how systems biology modeling offers strategies toward better understanding of drug synergy, including the protein-protein interaction (PPI) network-based methods, pathway dynamic simulations, synergy network motif recognitions, integrative drug feature calculations, and “omic”-supported analyses. Although partially successful in drug synergy exploration and interpretation, more efforts should be put on a holistic understanding of drug-disease interactions, considering integrative pharmacology and toxicology factors. With a comprehensive and deep insight into the mechanism of drug synergy, systems biology opens a novel avenue for rational design of effective drug combinations.

## Introduction

Drug combinations have been widely used in treating complex diseases such as cancer, HIV and cardiovascular diseases. For example, cisplatin and sabarubicin are co-administrated in lung cancer [1], combination of aspirin and dipyridamole is used to reduce the risk of stroke [2], and a novel FDA-approved drug combination: *Stribild*^TM^ [3] will been applied in the HIV-1 treatment. According to the Drug Combination Database (DCDB) [4], there have been 330 FDA-approved and 1033 investigational drug combinations until the second version. With more and more researches on this new form of therapy, drug combinations have been proven a promising way to combat complex diseases [5–9].

The most prominent benefit of a drug combination is the synergy effect among different drugs, where synergy means that the overall therapeutic effect of the combination is greater than the sum of effects caused by individual components [10]. In order to discover new combinations, quantification of drug synergy is one of the essential works. However, synergy quantification is not an easy task. Different dose–response methods, including Loewe additivity [11, 12], Bliss independence [13], and Chou-Talalay method [14] have been proposed. The main concept of these methods is to find out whether the observed combination effect or response departs from the expected effect, as reviewed in [10, 15, 16]. The dose–response methods play key roles in synergy quantification. However, no single method can describe synergy under all possible conditions. Besides, none of these methods has the overwhelming superiority under all conditions [10]; different forms of dose–response methods may even produce different results [17]. At present, it is still impractical to screen all possible drug combinations for different diseases. In addition, these dose–response methods cannot explain the potential synergy mechanism for drug combinations. Such problems suggest that the identification of synergistic drug combinations remains a non-trivial and challenging task. As “network-based drug discovery is taking the pharmaceutical industry into a new age” [18], systems biology and computational technologies have provided a powerful tool for multi-target drug discovery [19–21]. Consequently, systems biology has become a compensative way to predict novel synergistic drug combinations and to illustrate underlying mechanism. Such predictions can help accelerate the generation of hypotheses on possible synergistic drug combinations and guide the experimental conductions.

Systems biology allows us to describe the interactions among different molecules from a comprehensive network perspective. Currently, some systems biology approaches have been applied in drug synergy researches to predict the complex human body responses to drug combinations, to recognize the specific biological network features which are more likely to produce synergistic outcomes, and to illustrate the complicated molecular interactions which may contribute to synergistic mechanism [22–25]. Understanding the explicit relationships among different objects involved in drug combinations allows us to appreciate how drugs interact with each other to produce synergistic therapeutic effects.

In the future, systematic synergy modeling will provide an assistant tool independent of screening data to accelerate the generation of hypotheses and guide the experimental activities. Here we review the current status of this new field and describe how, in our view, systems biology models (as classified in Table 1) can help us to develop a predictive and mechanistic model of drug synergy.

**Table 1:** A classification of systematic drug synergy prediction models

Model type	Description	Inputs	Methods	Outputs
PPI network-based models	Evaluate drug synergy based on network topology relations of targets	Drug targets;	Complex network;	Synergistic drug combinations;
		Protein interactions	Network pharmacology	Synergistic targets
Pathway-based models	Simulate the dynamic changes of pathway and identify synergy-specific pathway structures	Drug targets;	Ordinary differential equations;	Dose–response assessment of drug synergy;
		Pathway structures;	Network motif recognition	Synergy-specific
		Dynamic changes of each pathway component;		network motifs in pathways
		Drug interactions		
Drug similarity-based models	Construct classification models based on various drug similarities	Drug properties like targets, structures, indication, et al.	Similarity calculation;	Synergistic drug combinations;
			Feature selection;	Distinctive features for drug combination
			Classification model	
Omic-based models	Apply “omic” data to calculate drug associations; or rebuild the synergy-dependent pathways	Responses to drugs in the form of “omic” data	Reverse engineering of biological networks;	Synergistic drug combinations;
			“Omic” data similarity calculation;	Biological networks;
			Classification model	

## Review

### Predicting synergy combinations based on the PPI network

The traditional “one drug, one target” therapeutic mode can only yield limited effects on complex diseases, because these diseases are mostly controlled by complex biological networks which involve compensative biological processes [26, 27]. On the contrary, drug combinations, with multiple targets belonging to interlinked processes, can combat the systematic pathological states through the cooperative mechanism. Researchers have recently applied network-based systems biology approaches to predict potential synergistic drug combinations, as well as to reveal the synergy mechanism from the perspective of biological molecular network. Most of such researches based on the hypothesis that synergistic drugs tend to have effects on targets with distinguishing network topological features [28–31].

**Fig. 1:**
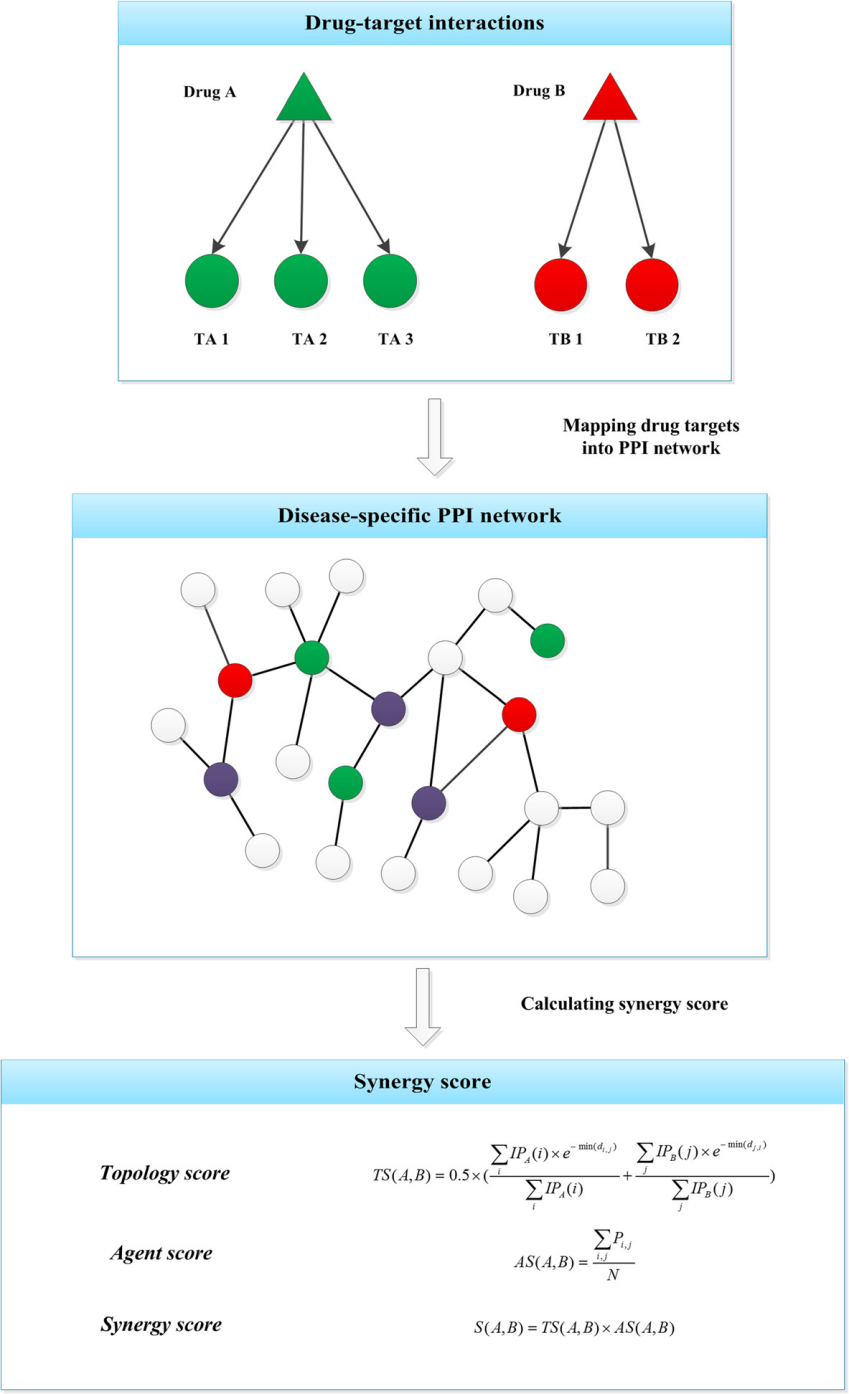
A schematic example of the PPI network-based synergy score calculation. The calculation strategy in this example was from the method described in [28]. The green and red triangles represent two different drugs. Each drug is targeting on certain protein targets denoted as circles with the same color as the corresponding drug. All the targets are mapped into a disease-specific PPI network, in which purple circles are pathological proteins, while white circles are other high-related proteins, and different proteins are connected by PPIs. Based on the disease-specific PPI network (*G*), topology score *TS(A, B)* can be calculated by the topology connection and centrality for targets of drug A (*TA*) and targets of drug B (*TB*), where *IP* means a integrative topological parameter calculated by integrating betweenness, closeness and a variant of the eigenvector PageRank through PCA, *IP*_*A*_ *(i)* is calculated for the target *i* of drug A and *IP*_*B*_ *(i)* for the target *j* of drug B, and *min(d*_*i,j*_ *)* represents the minimum shortest path from the *i*th target of drug A to all targets of drug B. Another agent score (*AS*), in which *P*_*i,j*_ represents the similarity score between the *i*th phenotype of drug A and the *j*th phenotype of drug B, can also be coupled with to adjust the topology-based score. At last, a synergy score can be calculated by the product of *TS* and *AS*

One of the most frequently used biological molecular networks is the protein-protein interaction (PPI) network which is based on intentional physical or functional associations between proteins. The PPIs are the core of the whole interactomics system of any living organism and are also the foundation of more highly specified networks such as a disease-specific molecular network. The disease-specific PPI network often refers to a protein network in which the proteins are related with one certain disease [32–34]. Based on a disease-specific PPI network, a heuristic synergy score can be calculated based on the topology connection and centrality of drug targets to predict the possible synergy degree. Other associated scores can also be coupled with to adjust the topology-based scores. For example, two evaluation scores, topology score (TS) and agent score (AS) were created to evaluate drug synergy for two drug combination by an algorithm termed NIMS (network target-based identification of multicomponent synergy) [28] (as outlined in Fig. 1). The TS was based on the topology relationship among targets from different components in the context of a previously defined disease-specific PPI network. The TS for two drugs will be high if they target on proteins which are close to each other on the PPI network and with high network centralities. The AS was to measure the phenotype similarity between the corresponding phenotypes of drugs. This phenotype similarity quantifies the overlap of their OMIM descriptions and is calculated by a text mining method. At last, a predictive synergy score was calculated as the product of TS and AS. Some synergistic ingredients from anti-angiogenic traditional Chinese medicine were successfully recognized by this score. Likewise, in the background of a disease-specific PPI network integrated of different data sources, Vitali et al. [29] assigned a score called Topological Score of Drug Synergy (TSDS) to evaluate the multi-target combination effects based on the target topological features including node reachability, global effect and synergistic effect. This work has been applied to identify novel targets which may lead to synergistic outcomes for Type 2 Diabetes Mellitus. To cover more knowledge about drug behavior, Huang et al. [30] proposed another synergy evaluation tool: “DrugComboRanker”. In this approach, the synergistic score incorporated topological relatedness of targets in the signaling network, semantic similarity of gene ontologies, and dissimilarity of the gene expression profiles of different drugs. The method was assessed on lung adenocarcinoma and endocrine receptor positive breast cancer and most of the top-ranked synergistic combinations were confirmed by literature reports.

PPI network-based method is an effective and straightforward way to elucidate the inter-relationship between a complex disease and drug interventions. In a generous way, Wang et al. [31] have indicated that drug combinations tend to target proteins which are closer in the genetic interaction networks comparing against random combinations. In network-based synergy evaluation, target acquisition and disease network construction are two basic steps. By mapping drug targets onto disease-related molecular networks, it can not only help reveal the mechanisms of action for each drug, but also highlight how different drugs in a combination cooperate with each other to achieve synergy effects. The corresponding topology relationships of drug targets are of importance for estimating synergy among combinations. However, it is a fact that the behavior of a drug is not only determined by its targets; other factors, like drug side effects, drug-resistance and other pharmacokinetic characteristics, will also be the root of drug synergy. Therefore, a more comprehensive model which is not limited to PPIs should be constructed to model the complicated interactions involved in the mechanism of action for drug combination.

### Exploring synergy mechanism from the perspective of pathway

A pathway refers to a series of chemical reactions or molecular interactions within a cell that generate a specific product, response or change in the cell [35]. Although the PPI network-based approaches described above can help discover potential synergistic drug combinations, pathways, which can represent specific parallel, cross-talk or feedback structures of molecular networks, are even more powerful for the explanation of synergy mechanism in more details. Different drugs can have influences on the same or different pathways, and induce synergy effects produced by targets aggregating at specific pathway structures, as revealed in [36]. Modeling the dynamic changes and network structures of pathways can help improve our understanding on the potential synergy mechanism for drug combinations.

### Dynamic pathway simulation

**Fig. 2:**
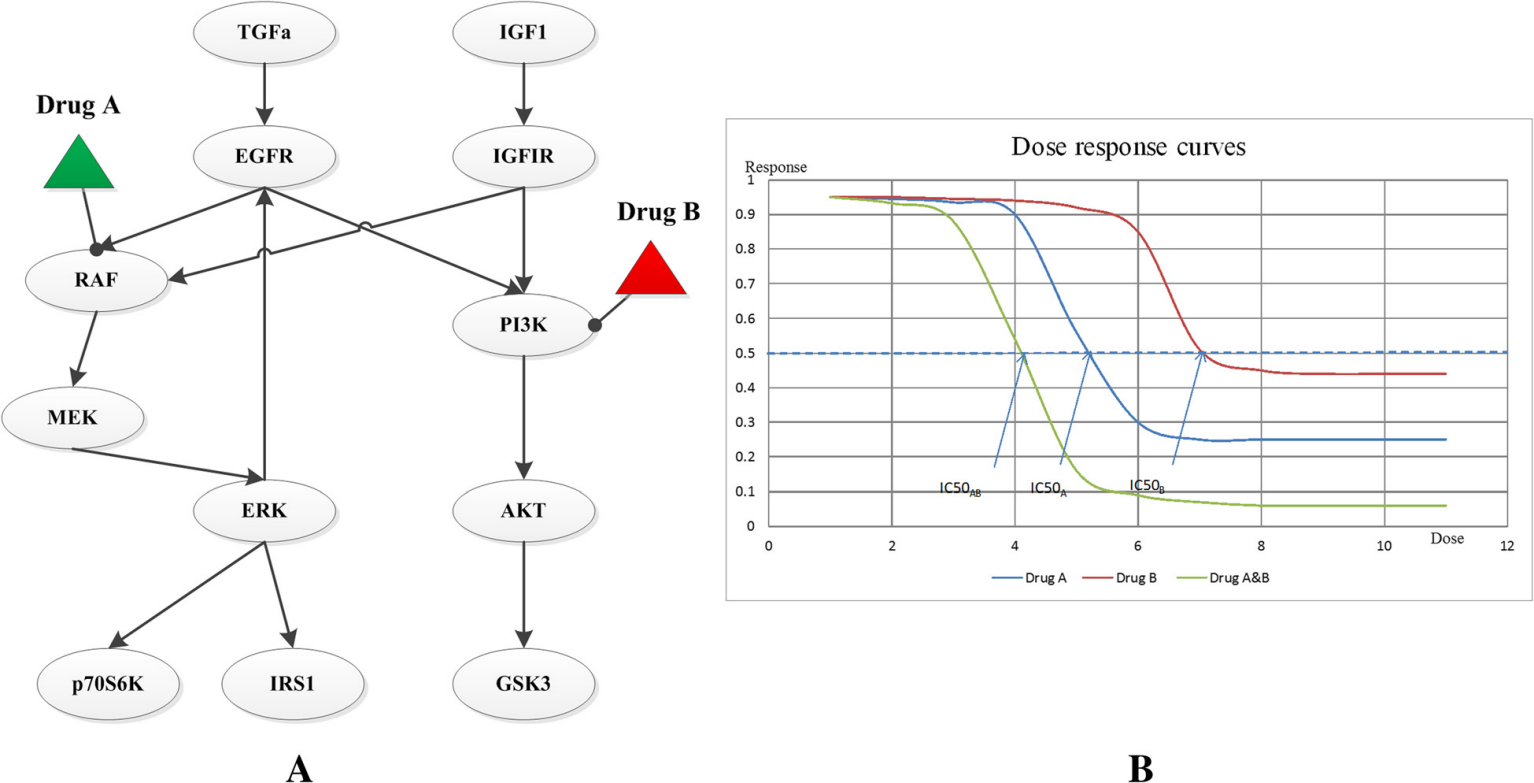
An example of pathway simulation model. **a**. A network-based pathway simulation model of the EGFR signaling pathway. Circles represent different components in a pathway. Triangles are drugs. Edges represent the reactions among different elements of the pathway, and the quantitative dynamic changes for each element can be described by one ODE or other computational models. This pathway network is adapted from Klinger et al. [44]. **b**. Dose–response plots. The dose–response curves are based on the pathway simulation results for each individual drug (a, b) and the drug combination (ab) respectively, where the half maximal inhibitory concentration (IC50) can be utilized to quantitatively assess drug synergism by dose–response methods like Loewe additivity. If the ratios of two drugs in the combination are both 0.5, an interaction index can be calculated as: $\frac{0.5\times IC{50}_{AB}}{IC{50}_A}+\frac{0.5\times IC{50}_{AB}}{IC{50}_B}$, where the denominators represent doses of each drug in the combination that yield a half inhibition effect, while the numerators represent the dose of each drug to produce the same effect when given alone. If this index is larger than one, two drugs are antagonistic, if it is less than one, the combination is synergistic. For the given example, this index is less than one, so drug A and B are synergistic

Dynamic simulation of specific pathway under drug interferences can help us understand the dynamic behavior of drugs, and provide insights into the synergy mechanism of certain drug combinations. Such pathway simulation models are often represented in a network form: nodes stand for concentrations or activity levels of pathway components (e.g., gene, protein or metabolite) and edges reflect the interaction of one node on the time derivative of another (Fig. 2a). Moreover, dose–response data can be obtained from the simulation results (Fig. 2b). Based on these results, synergy can be quantified by traditional dose–response methods like Bliss independence and Loewe additivity.

Classical chemical kinetics utilizes ordinary differential equations (ODE) to describe system dynamics [37–39]. A system of ordinary differential equations is the most fundamental way to quantitatively simulate the dynamic response of each individual molecule in a pathway under different conditions like the interventions of drug combinations [40–43]. Based on a kinetic ODEs model of the epidermal growth factor receptor (EGFR) signaling pathway, Araujo et al. [40] simulated the effects of combinatorial kinase inhibitors and discovered that a simultaneous inhibition of multiple nodes in a signaling cascade with small molecule kinase inhibitors will be a new promising combination strategy for cancer treatment. Modification of classical kinetic ODE models can improve the ability in reproducing cellular dynamics. Nelander et al. [41] derived a non-linear ODE model to capture the important phenomena which cannot be described by simple linear model, like the epistasis and saturation effects in a cellular system. Based on this model, the authors can predict the quantitative outcomes of combinatorial perturbations in breast cancer cells. Likewise, Miller et al. [43] also utilized a non-linear ODE model to quantitatively simulate the dynamic changes of signaling pathways in dedifferentiated liposarcoma cells. Integrating with the experimental drug combination screening, the simulation helps to explain that the synergy of CDK4 and IGF1R inhibitors may depend on the activity of AKT pathway.

Except for the ODE models, other computational methods have also been applied in pathway modeling for drug combination analysis. A modeling framework based on the modular response analysis (MRA) was developed to build a simulation model for the EGFR signaling pathway [44]. The MRA model can quantitatively simulate the dynamic changes triggered by feedback cycle, feedforward loop as well as cross talk structures in pathways. Based on the MRA model, Klinger et al. [44] deduced that downstream mutations do not necessarily invalidate upstream drug treatment if a suitable downstream inhibitor were combined. Different researches pay attention to different types of pathway structures. Yan et al. [45] proposed a mathematical model to simplify the serial and parallel structures of biological pathways. Besides, Asfar et al. [46] utilized the network modeling based on the Ingenuity Pathway Anallysis (IPA) Software which considers the microarray datasets at different time points to understand central synergy mechanisms for the combination of MI219 and oxaliplatin. Sometimes, simulations only based on the disease-specific pathway are not enough to describe the drug synergy mechanism, extra pathways which can indirectly affect the therapeutic process should also be considered. In the study of Li et al. [47], which aimed to investigate on the role of immune system response in mediating anti-influenza drug synergy, both of the influenza A virus life cycle pathway and two immune-related pathways were simulated by a series of delay differential equations.

### Synergy-specific network motifs in pathways

**Fig. 3:**
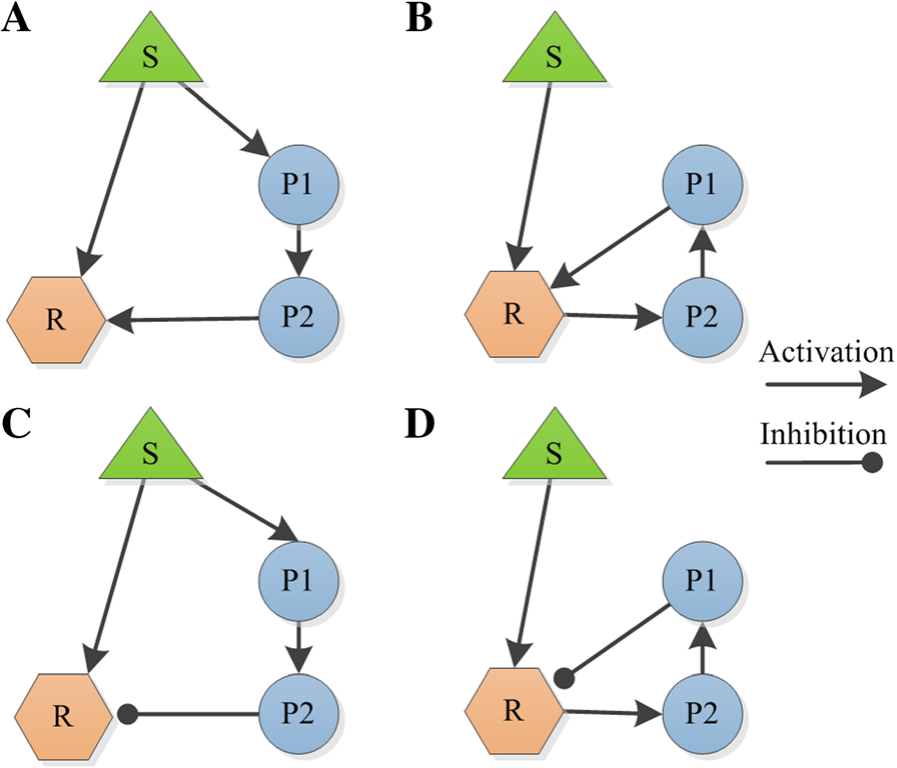
Examples of network motifs in pathways. **a**. Positive feedforward; **b**. Positive feedback; **c**. Negative feedforward; **d**. Negative feedback. S represents stimulus, P1, P2 are proteins in a pathway, and R represents the effector protein. Different types of the directed edges represent activation or inhibition effects between two connected elements. For example, P1 has an activation effect on P2 in Fig. A, while P1 has an inhibition effect on R in Fig. D. The study of Zhang et al. [53] discovered that combinations may be more probably to exhibit synergistic effects when targeting on a negative feedback motif. The study of Yin et al. [54] found antagonistic drug interactions are mostly related with a positive feedback loop. This figure is reproduced from [50], with permission from Elsevier (RightsLink license number: 3687391088768)

The above-mentioned pathway simulation models provide a more concrete way to predict effective drug combinations. What’s more, they offer tools to discover which types of structures in pathways are more inclined to generate drug synergies, thus illustrating the mechanism of synergy at the pathway level. However, the pathway simulation models are often hampered by their incompleteness and complexity. To address this problem, reduced or abstract network models which can capture key dynamical properties of a network can be used. These reduced models, termed as network motifs, are composed of same number of elements and are used to describe distinct connectivity patterns that occur frequently in the whole network or different pathways [48–50] (Fig. 3). Previous researches have begun to depict the dynamics of network motifs by kinetic ODE methods, and describe how variations in the kinetic parameters affect the human body responses to drug interventions [50–52]. Different studies may produce distinctive forms of motifs considering their specific focuses. In the study of Zhang, et al. [53], based on ten three-node small network motifs covering main pathway sub-structures like positive feedback loop, negative feedback loop, positive auto-regulation, and feed-forward loop, they recognized which kind of concurrent variations could act synergistically (or antagonistically) to alter the responses of the motifs and which type of motifs were more likely to lead to synergistic (or antagonistic) responses. They discovered that combinations may be more probably to exhibit synergistic effects when targeting on a negative feedback loop motif or a mutual inhibition loop motif. As another example, Yin et al. [54] used three-node enzymatic motifs to study about the synergistic or antagonistic interactions within drug combinations considering different motif topologies and parameters. This study found that drug combination effects largely depend on network topology, and the kinetic parameter variations only yield limited influences on the drug combination effects. The specific synergistic or antagonistic network motifs for drug combinations were identified: synergistic motifs often encompass serial and parallel or mix-type structures, while antagonistic motifs are mostly with a positive feedback loop and a downstream link.

Although the pathway or network motif simulation results may not fully recreate the absolutely real biological phenomena, considering the network structures of the pathways which are more inclined to generate synergistic effects, these models have also come out with some hypotheses about the synergy mechanism underlying drug combinations: drugs targeting on certain types of structures, like cross-talk, parallel or mutual inhibition loop in pathways will lead to synergistic drug combination effects. Although these hypotheses may not hold true in all cases, they can still provide guidance for the rational design of potentially effective drug combinations. Some studies can reduce the combinatorial drug screening space by selecting drugs with targets on compensatory or inter-linked pathways [55–57].

However, one limitation of these pathway models is that we only partially understand the pathways of most diseases or drugs, as pointed out in [58]. With the accumulation of the “omic” data and various reverse engineering methods [59, 60], more and more disease-specific pathways can be reconstructed. These new pathways will provide more bases for drug synergy mechanism illustration. However, most of the existing researches focus on the target-pathway (or motifs) interactions, ignoring other drug-organism interactions (e.g., drug absorption or metabolism). To obtain a more comprehensive description of synergy mechanism, future studies should pay more attention to construction of comprehensive network models considering not only drug targeting pathways but also other drug behaviors like absorption, metabolism, transportation, and even toxicity-related pathways.

### Computational models based on integrated drug similarities

In addition to the network-based synergy principles, other forms of synergy mechanism, which can be owed to certain chemical or pharmacological properties such as chemical structure, drug indication, and drug side effects, should also be characterized. In order to understand drug synergy from a more global perspective, different types of drug properties need to be considered simultaneously. Some computational methods have been proposed to integrate various similarities based on different drug properties to build classification models for drug combinations.

Drugs or drug combinations can be represented as their corresponding features described by multiple drug properties. Thereby different machine learning methods can be utilized in the prediction or classification for synergistic drug combinations. For example, Zhao et al. [61] developed a computational model for the prediction of effective drug combinations based on the correlations between candidate combinations and confirmed combinations. In this study, each drug pair was featured as the pairwise combinations of their comprehensive molecular and pharmacological elements, including drug targets and their corresponding downstream pathways, medical indication areas, Anatomical Therapeutic Chemical (ATC) codes, and side effects. Based on the enrichment levels of the candidate drug pair features among all the drug pair features from known drug combinations, Zhao et al. [61] have predicted novel pairwise drug combinations, among them 69 % can be validated by literature reports. Another research used associations of drugs in a combination to describe drug combination features [62]. Considering three different kinds of drug relationships including chemical interactions between drugs in the combination, protein interactions between the targets of drugs, and target enrichment of Kyoto Encyclopedia of Genes and Genomes (KEGG) pathways, each drug combination was represented as a high-dimensional numeric vector. Then a random forest classification model was constructed based on the key features extracted from high-dimensional numeric vectors to predict synergistic drug combinations. A 5-fold crossover validation showed this classification model was well performed with a Matthews correlation coefficient of 0.673.

Prediction or classification models based on properly selected drug properties can help to identify potential synergistic drug combination. The most important part of such computational models is the feature calculation for drug combinations. Informative features will improve the prediction accuracy and help to reveal synergy mechanism. Besides, samples collection is another important step. Consequently, more efforts should be put in the elaborate design of effective features for identification of drug combinations and collection of drug combinations with or without confirmed synergistic effects.

### “Omic”-based methods in synergy identification

With advances in bioinformatics, systems biology and molecular biology, different types of high-throughput “omic” data such as genomics, transcriptomics and metabolomics data have emerged as critical information in the biomedical and pharmaceutical fields [63–65]. The “omic” data have been widely used in the establishment of interactions among small molecules, genes and diseases and the identification of molecules specifically associated with certain pathological processes [66–69]. Owing to its benefits in reflecting biological responses, “omic” data has also made great contributions to drug combination studies. One of the pioneering studies was the CMAP project by Lamb et al. [69]. Based on this project, the researchers illustrated the possibility of rational drug combinations [33, 69].

Considering the aim of “omic” is at collectively quantifying pools of biological molecules [70], specific computational approaches has also been proposed to predict the human-body responses to drug combinations or constructing novel molecular networks based on “omic” data [33, 71–74].

The most commonly used “omic” data are the genomic data. To reduce the burden in genomic data acquisition for all possible combinations, the corresponding genomic data for drug combination has been predicted by computational methods based on the data of individual ones. Based on this principle, Wu et al. [71] proposed a computational scheme to predict the gene expression profiles for drug combinations based on gene profiles treated with individual drugs. Then, according to the differential expression changes of genes, they applied a new integer programming model to identify sub-networks affected by single drugs or combinations from the background molecular network which included protein interactions, protein-DNA interactions, and signaling pathways. Finally, the best candidates of combination drugs were determined by taking into account efficacy and side-effect based on the identified sub-networks. The genomic data can also help reconstruct specific molecular network. As mentioned above, the synergy ranker: “DrugComboRanker” [33] utilized the genomic data for drug functional network construction. Moreover, the Petri-net model which is a graph-based mathematical method for the description of distributed systems has also been applied in drug combination analysis based on the genomic data. Jin et al. [72] proposed an enhanced Petri-net (EPN) model based on the gene expression data to simulate drug effects. According to the EPN model, the synergistic effects of pairwise drug combinations were identified when there was at least one molecule that shows an enhanced effect in the combination comparing with the summation effects of two individual drugs; and the researchers illustrated that the synergy mechanism was related with the feedback loops among molecules. Besides, some researchers adopted correlation-based strategies to calculate the similarities of “omic”-based responses to drug interventions [73, 74]. Based on the genome-wide expression profiles, Zhao et al. [73] have revealed the synergistic drug effects by quantitatively profiling cellular responses to drugs. According to the drug similarities based on a chemogenomic profile, Jansen et al. [74] identified antifungal synergies. They found that compound pairs that have correspondingly similar profiles are more likely to be synergistic when compared with randomly chosen compounds.

In addition to genomic data, proteomics is another type of “omic” data for drug synergy study. This type of data can help reconstruct the signaling pathways. As we have mentioned in “Dynamic pathway simulation”, the signaling pathways were reconstructed based on the phosphorylation data of key signaling proteins [40–46] by different pathway simulation methods.

Integration of different types of “omic” data can also help explore synergy mechanism. By incorporating phosphoproteomics, transcriptomics and chemical proteomics data, Winter et al. [75] discovered that the underlying synergy mechanism for the combination of danusertib and bosutinib in the treatment of imatinib-resistant Chronic myelogenous leukemia (CML) might be related with the nonobvious off target effects implicated in the Mitogen-activated protein kinases (MAPK) signaling pathway.

With accumulation of “omic” data, the field of systems biology has progressed at an even higher speed. The “omic”-based drug synergy studies are presently mainly conducted on the genomics and proteomics levels. To understand the complex interplay of drug combinations against the disease process, integrative “omic”-based models covering across various “omic” data like genetic, transcriptomic, proteomic and metabolomics data need to be developed.

## Conclusion

Rational design of drug combinations with synergistic effects remains a challenging task despite tremendous experimental and clinical efforts. Examples presented in this review illustrate how systems biology approaches are making encouraging contributions to explore synergistic drug combinations. Given the complexity of synergy mechanism and the difficulty in drug combination screening, systematic synergy modeling will become a very important component in the field of drug combinations beyond question. However, there are still great challenges to overcome.

Network or pathway-based methods have already facilitated the prediction of synergistic drug combinations and promoted the understanding of synergy mechanism. Based on varied pathway modeling methods and the increasing “omic” data, pathways for different diseases can be reconstructed, thus providing firm foundations for the exploration of synergy. However, mostly focusing on drug targets to represent drug action, these models for drug synergy mainly depend on the interaction between targets and disease-specific network or pathways. Such methods neglect the fact that synergy can also arise when one drug increases the effects of other drugs by influencing on their certain properties like bioavailability as revealed in [76]. Drug behaviors are not simply determined by targets, other factors like the absorption, distribution, metabolism, and excretion (ADME) processes and off-target activities can also have significant impacts on drug therapeutic outcomes. Even though some classification models based on integrated drug features considering comprehensive chemical and pharmacological properties have overcome this “target-only” issue; however, the current number of effective drug combinations and their obtainable properties will limit the performance. Consequently, more attention should be paid on the acquisition and integration of extensive knowledge to model the drug-organism interplay.

Systems biology models for drug synergy evaluation are still in their infancy, but have shown prominent advantages in exploring synergistic drug combinations. The potential synergy mechanism has also been illustrated based on these methods. To understand drug synergy from a more comprehensive perspective, future efforts should be put on novel approaches which can model the synergistic effects from a more holistic perspective covering across pharmacology-considering both pharmacodynamics and pharmacokinetics elements-and toxicology spaces. With a deep insight into drug synergy, systems biology approaches will serve as a compensative tool for the rational design of effective drug combinations.

However, it should also be noted that systems biology modeling is only an aided tool but not the judge. The drug synergy predictions can provide valuable references for experimental drug combination screening, but they cannot substitute the experimental methods to determine the final statement on drug synergism or antagonism. The systematic synergy modeling methods can provided assistances for drug synergy researches, but the effectiveness and accuracy of these predictive models should still rely on experimental evaluations.

## Abbreviations

PPI: Protein-protein interactions
FDA: Food and Drug Administration
DCDB: Drug Combination Database
TS: Topology Score
AS: Agent Score
NIMS: Network target-based Identification of Multicomponent Synergy
TSDS: Topological Score of Drug Synergy
CMAP: Connectivity Map
ODE: Ordinary Differential Equations
EGFR: Epidermal Growth Factor Receptor
MRA: Modular Response Analysis
IC50: half maximal Inhibitory Concentration
IPA: Ingenuity Pathway Anallysis
ATC: Anatomical Therapeutic Chemical
KEGG: Kyoto Encyclopedia of Genes and Genomes
EPN: Enhanced Petri-Net
CML: Chronic myelogenous leukemia
MAPK: Mitogen-activated protein kinases
ADME: Absorption, Distribution, Metabolism, and Excretion
PCA: Principal Component Analysis

## References

[1] Bigioni M, Benzo A, Irrissuto C, Lopez G, Curatella B, Maggi CA (2008). Antitumour effect of combination treatment with Sabarubicin (MEN 10755) and cis-platin (DDP) in human lung tumour xenograft. Cancer Chemother Pharmacol..

[2] Lenz TL, Hilleman DE (2000). Aggrenox: a fixed-dose combination of aspirin and dipyridamole. Ann Pharmacother..

[3] Breeze S (2012). Novel HIV-1 treatment Stribild gains regulatory approval. Expert Rev Clin Pharmacol..

[4] Liu Y, Hu B, Fu C, Chen X (2010). DCDB: drug combination database. Bioinformatics..

[5] Albers GW, Amarenco P (2001). Combination Therapy With Clopidogrel and Aspirin. Stroke..

[6] Bezin J, Pariente A, Lassalle R, Dureau-Pournin C, Abouelfath A, Robinson P (2014). Use of the recommended drug combination for secondary prevention after a first occurrence of acute coronary syndrome in France. Eur J Clin Pharmacol..

[7] Singh S, Bouzinbi N, Chaturvedi V, Godreuil S, Kremer L (2014). In vitro evaluation of a new drug combination against clinical isolates belonging to the Mycobacterium abscessus complex. Clin Microbiol Infect..

[8] Lee HZ, Miller BW, Kwitkowski VE, Ricci S, DelValle P, Saber H (2014). U.S. Food and drug administration approval: obinutuzumab in combination with chlorambucil for the treatment of previously untreated chronic lymphocytic leukemia. Clin Cancer Res.

[9] Patankar NA, Pritchard J, van Grinsven M, Osooly M, Bally MB (2013). Topotecan and doxorubicin combination to treat recurrent ovarian cancer: the influence of drug exposure time and delivery systems to achieve optimum therapeutic activity. Clin Cancer Res..

[10] Sucher NJ (2014). Searching for synergy in silico, in vitro and in vivo. Synergy.

[11] Loewe S (1927). Die Mischiarnei. Klin Wochenschr..

[12] Loewe S (1953). The problem of synergism and antagonism of combined drugs. Arzneimittelforschung..

[13] Bliss CI (1939). The toxicity of poison applied jointly. Ann Appl Biol..

[14] Chou TC (2010). Drug combination studies and their synergy quantification using the Chou-Talalay method. Cancer Res..

[15] Chou TC (2006). Theoretical basis, experimental design, and computerized simulation of synergism and antagonism in drug combination studies. Pharmacol Rev..

[16] Tallarida RJ (2011). Quantitative methods for assessing drug synergism. Genes & cancer..

[17] Doern CD (2014). When does 2 plus 2 equal 5? A review of antimicrobial synergy testing. J Clin Microbiol..

[18] Leung EL, Cao ZW, Jiang ZH, Zhou H, Liu L (2013). Network-based drug discovery by integrating systems biology and computational technologies. Brief Bioinform..

[19] Csermely P, Agoston V, Pongor S (2005). The efficiency of multi-target drugs: the network approach might help drug design. Trends Pharmacol Sci..

[20] Lu JJ, Pan W, Hu YJ, Wang YT (2012). Multi-target drugs: the trend of drug research and development. PLoS One.

[21] Schrattenholz A, Groebe K, Soskic V (2010). Systems biology approaches and tools for analysis of interactomes and multi-target drugs. Methods Mol Biol..

[22] Feala JD, Cortes J, Duxbury PM, Piermarocchi C, McCulloch AD, Paternostro G (2010). Systems approaches and algorithms for discovery of combinatorial therapies. Wiley Interdiscip Rev Syst Biol Med..

[23] Galizzi JP, Lockhart BP, Bril A (2013). Applying systems biology in drug discovery and development. Drug Metabol Drug Interact..

[24] Li P, Huang C, Fu YX, Wang JA, Wu ZY, Ru JL, et al. Large-scale exploration and analysis of drug combinations. Bioinformatics. 2015;31:2007–16. 10.1093/bioinformatics/btv080 25667546

[25] Berg EL (2014). Systems biology in drug discovery and development. Drug Discov Today..

[26] Zimmermann GR, Lehar J, Keith CT (2007). Multi-target therapeutics: when the whole is greater than the sum of the parts. Drug Discov Today..

[27] Li Y, Agarwal P. A Pathway-Based View of Human Diseases and Disease Relationships. Plos ONE. 2009;4:e4346. 10.1371/journal.pone.0004346 PMC2631151 19194489

[28] Li S, Zhang B, Zhang N (2011). Network target for screening synergistic drug combinations with application to traditional Chinese medicine. BMC Syst Biol.

[29] Vitali F, Mulas F, Marini P, Bellazzi R (2013). Network-based target ranking for polypharmacological therapies. J Biomed Inform..

[30] Huang L, Li F, Sheng J, Xia X, Ma J, Zhan M (2014). DrugComboRanker: drug combination discovery based on target network analysis. Bioinformatics..

[31] Wang YY, Xu KJ, Song J, Zhao XM (2012). Exploring drug combinations in genetic interaction network. BMC bioinformatics.

[32] Ren G, Liu Z (2013). NetCAD: a network analysis tool for coronary artery disease-associated PPI network. Bioinformatics..

[33] Wan FC, Cui YP, Wu JT, Wang JM, -Z Liu Q, Gao ZL (2013). The PPI network and cluster ONE analysis to explain the mechanism of bladder cancer. Eur Rev Med Pharmacol Sci.

[34] Wu B, Xie J, Du Z, Wu J, Zhang P, Xu L (2014). PPI network analysis of mRNA expression profile of ezrin knockdown in esophageal squamous cell carcinoma. Biomed Res Int..

[35] Viswanathan GA, Seto J, Patil S, Nudelman G, Sealfon SC (2008). Getting started in biological pathway construction and analysis. PLoS Comput Biol..

[36] Jia J, Zhu F, Ma X, Cao Z, Li Y, Chen YZ (2009). Mechanisms of drug combinations: interaction and network perspectives. Nat Rev Drug Discov..

[37] Polynikis A, Hogan SJ, di Bernardo M (2009). Comparing different ODE modelling approaches for gene regulatory networks. J Theor Biol..

[38] Elias J, Dimitrio L, Clairambault J, Natalini R (2014). The dynamics of p53 in single cells: physiologically based ODE and reaction–diffusion PDE models. Phys Biol..

[39] Khan FM, Schmitz U, Nikolov S, Engelmann D, Putzer BM, Wolkenhauer O (1844). Hybrid modeling of the crosstalk between signaling and transcriptional networks using ordinary differential equations and multi-valued logic. Biochim Biophys Acta..

[40] Araujo RP, Petricoin EF, Liotta LA (2005). A mathematical model of combination therapy using the EGFR signaling network. Biosystems..

[41] Nelander S, Wang W, Nilsson B, She QB, Pratilas C, Rosen N (2008). Models from experiments: combinatorial drug perturbations of cancer cells. Mol Syst Biol..

[42] Sun X, Bao J, Nelson KC, Li KC, Kulik G, Zhou X (2013). Systems Modeling of Anti-apoptotic Pathways in Prostate Cancer: Psychological Stress Triggers a Synergism Pattern Switch in Drug Combination Therapy. PLoS Comput Biol..

[43] Miller ML, Molinelli EJ, Nair JS, Sheikh T, Samy R, Jing X (2013). Drug synergy screen and network modeling in dedifferentiated liposarcoma identifies CDK4 and IGF1R as synergistic drug targets. Sci signal.

[44] Klinger B, Sieber A, Fritsche-Guenther R, Witzel F, Berry L, Schumacher D (2013). Network quantification of EGFR signaling unveils potential for targeted combination therapy. Mol Syst Biol..

[45] Yan H, Zhang B, Li S, Zhao Q (2010). A formal model for analyzing drug combination effects and its application in TNF-alpha-induced NFkappaB pathway. BMC Syst Biol..

[46] Azmi AS, Banerjee S, Ali S, Wang Z, Bao B, Beck FWJ (2011). Network Modeling of MDM2 Inhibitor-Oxaliplatin Combination Reveals Biological Synergy in wt-p53 solid tumors. Oncotarget.

[47] Li Z, Zhou H, Lu Y, Colatsky T (2014). A Critical Role for Immune System Response in Mediating Anti-influenza Drug Synergies Assessed by Mechanistic Modeling. CPT Pharmacometrics Syst Pharmacol..

[48] Milo R, Shen-Orr S, Itzkovitz S, Kashtan N, Chklovskii D, Alon U (2002). Network motifs: simple building blocks of complex networks. Science..

[49] Wong E, Baur B, Quader S, Huang CH (2012). Biological network motif detection: principles and practice. Brief Bioinform..

[50] Han Z, Vondriska TM, Yang L, Robb MacLellan W, Weiss JN, Qu Z (2007). Signal transduction network motifs and biological memory. J Theor Biol..

[51] Tyson JJ, Novak B (2010). Functional motifs in biochemical reaction networks. Annu Rev Phys Chem..

[52] Fioravanti F, Helmer-Citterich M, Nardelli E (2012). Modeling gene regulatory network motifs using Statecharts. BMC bioinformatics.

[53] Zhang Y, Smolen P, Baxter DA, Byrne JH (2014). Computational analyses of synergism in small molecular network motifs. PLoS Comput Biol..

[54] Yin N, Ma W, Pei J, Ouyang Q, Tang C, Lai L (2014). Synergistic and Antagonistic Drug Combinations Depend on Network Topology. PLoS ONE..

[55] Axelrod M, Gordon VL, Conaway M, Tarcsafalvi A, Neitzke DJ, Gioeli D (2013). Combinatorial drug screening identifies compensatory pathway interactions and adaptive resistance mechanisms. Oncotarget..

[56] Dent P, Curiel DT, Fisher PB, Grant S (2009). Synergistic combinations of signaling pathway inhibitors: Mechanisms for improved cancer therapy. Drug Resist Updat..

[57] Azmi AS, Wang Z, Philip PA, Mohammad RM, Sarkar FH (2010). Proof of concept: network and systems biology approaches aid in the discovery of potent anticancer drug combinations. Mol Cancer Ther..

[58] Reddy AS, Zhang S (2013). Polypharmacology: drug discovery for the future. Expert Rev Clin Pharmacol..

[59] Parikh JR, Klinger B, Xia Y, Marto JA, Bluthgen N (2010). Discovering causal signaling pathways through gene-expression patterns. Nucleic Acids Res..

[60] Janes KA, Lauffenburger DA (2013). Models of signalling networks - what cell biologists can gain from them and give to them. J Cell Sci..

[61] Zhao XM, Iskar M, Zeller G, Kuhn M, van Noort V, Bork P (2011). Prediction of drug combinations by integrating molecular and pharmacological data. PLoS Comput Biol..

[62] Chen L, Li BQ, Zheng MY, Zhang J, Feng KY, Cai YD (2013). Prediction of effective drug combinations by chemical interaction, protein interaction and target enrichment of KEGG pathways. Biomed Res Int..

[63] Cui Y, Paules RS (2010). Use of transcriptomics in understanding mechanisms of drug-induced toxicity. Pharmacogenomics..

[64] Zhao S, Iyengar R (2012). Systems pharmacology: network analysis to identify multiscale mechanisms of drug action. Annu Rev Pharmacol Toxicol..

[65] Kaur G, Rajput B (2014). Comparative analysis of the omics technologies used to study antimonial, amphotericin B, and pentamidine resistance in leishmania. J Parasitol Res..

[66] Lee KJ, Yin W, Arafat D, Tang Y, Uppal K, Tran V (2014). Comparative transcriptomics and metabolomics in a rhesus macaque drug administration study. Front Cell Dev Biol..

[67] Stratton MR (2011). Exploring the genomes of cancer cells: progress and promise. Science..

[68] Tieri P, Zhou X, Zhu L, Nardini C (2014). Multi-omic landscape of rheumatoid arthritis: re-evaluation of drug adverse effects. Front Cell Dev Biol..

[69] Lamb J, Crawford ED, Peck D, Modell JW, Blat IC, Wrobel MJ (2006). The Connectivity Map: using gene-expression signatures to connect small molecules, genes, and disease. Science..

[70] Coughlin SS (2004). Toward a road map for global -omics: a primer on -omic technologies. AM J Epidemiol..

[71] Wu Z, Zhao XM, Chen L (2010). A systems biology approach to identify effective cocktail drugs. BMC Syst Biol.

[72] Jin G, Zhao H, Zhou X, Wong ST (2011). An enhanced Petri-net model to predict synergistic effects of pairwise drug combinations from gene microarray data. Bioinformatics..

[73] Zhao J, Zhang XS, Zhang S (2014). Predicting cooperative drug effects through the quantitative cellular profiling of response to individual drugs. CPT Pharmacometrics Syst Pharmacol..

[74] Jansen G, Lee AY, Epp E, Fredette A, Surprenant J, Harcus D (2009). Chemogenomic profiling predicts antifungal synergies. Mol Syst Biol..

[75] Winter GE, Rix U, Carlson SM, Gleixner KV, Grebien F, Gridling M (2012). Systems-pharmacology dissection of a drug synergy in imatinib-resistant CML. Nat Chem Biol..

[76] Cokol M, Chua HN, Tasan M, Mutlu B, Weinstein ZB, Suzuki Y (2011). Systematic exploration of synergistic drug pairs. Mol Syst Biol..

